# Anatomically Detailed and Large-Scale Simulations Studying Synapse Loss and Synchrony Using NeuroBox

**DOI:** 10.3389/fnana.2016.00008

**Published:** 2016-02-12

**Authors:** Markus Breit, Martin Stepniewski, Stephan Grein, Pascal Gottmann, Lukas Reinhardt, Gillian Queisser

**Affiliations:** ^1^Computational Neuroscience, Department for Computer Science and Mathematics, Goethe Center for Scientific Computing, Goethe UniversityFrankfurt am Main, Germany; ^2^Department of Mathematics, Temple UniversityPhiladelphia, PA, USA

**Keywords:** HPC, large-scale neuronal networks, synaptic plasticity, electrical scale, anatomy, reconstruction, simulation, cable equation

## Abstract

The morphology of neurons and networks plays an important role in processing electrical and biochemical signals. Based on neuronal reconstructions, which are becoming abundantly available through databases such as *NeuroMorpho.org*, numerical simulations of Hodgkin-Huxley-type equations, coupled to biochemical models, can be performed in order to systematically investigate the influence of cellular morphology and the connectivity pattern in networks on the underlying function. Development in the area of synthetic neural network generation and morphology reconstruction from microscopy data has brought forth the software tool NeuGen. Coupling this morphology data (either from databases, synthetic, or reconstruction) to the simulation platform UG 4 (which harbors a neuroscientific portfolio) and VRL-Studio, has brought forth the extendible toolbox NeuroBox. NeuroBox allows users to perform numerical simulations on hybrid-dimensional morphology representations. The code basis is designed in a modular way, such that e.g., new channel or synapse types can be added to the library. Workflows can be specified through scripts or through the VRL-Studio graphical workflow representation. Third-party tools, such as ImageJ, can be added to NeuroBox workflows. In this paper, NeuroBox is used to study the electrical and biochemical effects of synapse loss vs. synchrony in neurons, to investigate large morphology data sets within detailed biophysical simulations, and used to demonstrate the capability of utilizing high-performance computing infrastructure for large scale network simulations. Using new synapse distribution methods and Finite Volume based numerical solvers for compartment-type models, our results demonstrate how an increase in synaptic synchronization can compensate synapse loss at the electrical and calcium level, and how detailed neuronal morphology can be integrated in large-scale network simulations.

## 1. Introduction

The structure of neurons and networks in the brain is known to change continuously over time. Cellular growth, synapse formation or synapse loss, reorganization of intracellular architecture constantly make changes to the overall cellular and network anatomy (Hughes, 1958; Abbott and Nelson, 2000; Sheng and Hoogenraad, 2007; Shepherd and Huganir, 2007; Tai et al., 2008; Colon-Ramos, 2009; Branco et al., 2010; Zeltser et al., 2012; Tyagarajan and Fritschy, 2014). These changes in geometric layout can be interpreted as a strong indicator that the anatomy of the (sub)cellular and network level is deeply involved on various functional levels. Neuroscientific research has always been devoted to the interplay between morphology and function on various functional levels. Experimental research draws from microscopy techniques that can make morphology and spatio-temporal signals visible (Spacek and Harris, 1997; Arellano et al., 2007; Chen et al., 2008), theoretical work in Computational Neuroscience has brought forth an abundant spread of cellular and network models, many of them rely on a spatial representation of neurons and networks (Bower and Beeman, 1997; Hines and Carnevale, 1997; Balls et al., 2004; Gewaltig and Diesmann, 2007; Andrews et al., 2010). General purpose simulators such as NEURON or Genesis couple electrical and biochemical models to graph-representations of neurons and synaptically connected networks. The importance of neuronal morphology used in such simulations can be seen in reconstruction projects, such as the database project *NeuroMorpho* (cf. Ascoli, 2006). Currently more than 30,000 cell reconstructions are freely available on this platform.

Reconstructing morphology from microscopy data is a further example of how deeply structure is integrated in the brain. Semi-manual or fully automated reconstruction methods are being developed in research groups around the world (e.g., Jungblut et al., 2011; Popov et al., 2011; Burette et al., 2012), trying to unravel the filigreed multi-level organization of the brain. This dedication has advanced the field significantly, still many of the anatomical questions are currently unresolved. To leverage the power of large-scale network simulations, synthetic neuron morphology tools have been developed (Wolf et al., 2013). These algorithms are capable of generating synthetic networks with realistic morphology statistics which can be used within detailed functional simulations. In order to use these large data sets in detailed and large network simulations high performance computing platforms become an inevitable component of the process. While most of the available network simulators were originally conceived to run serially, there has been effort to parallelize and optimize the code for ever growing computing power.

In this paper, we present an approach focusing on the topic of cellular and network anatomy within a large-scale computing context. Building on scalable numerical methods in a flexible and parallelized discretization and solver framework for general ordinary and partial differential equation systems, this unified approach does not make use of the NEURON simulation environment (Hines and Carnevale, 1997) used in similar projects (Markram, 2015; Ramaswamy, 2015; Reimann, 2015). We introduce some of the authors' contributions in morphology reconstruction as well as artificial construction, hybrid-dimensional modeling and simulation of coupled biochemical and electrical signals, and link these to newly developed algorithms for massively parallel simulation of cable equation models and synapse distribution on cells. The latter can be used to simulate healthy and disease state neurons with different synapse numbers and distributions.

The Materials and Methods section of this paper discusses the tool NeuGen (Eberhard et al., 2006; Wolf et al., 2013) and how it ties into a generalized simulation framework. Our model for simulating electrical signals builds upon the known cable theory and is briefly summarized. We introduce our methods for handling synapse types and synapse distributions and introduce a new way of numerically discretizing the resulting model equations and computational domains, ultimately resulting in a system that can be solved on massively parallel computing architectures. These methods are compiled in the toolbox NeuroBox which is developed on top of the numerics engine UG 4 (cf. Vogel et al., 2013) that has been used in several detailed studies of structure-function interplay (Xylouris et al., 2007; Hansen et al., 2008; Nägel et al., 2008, 2009; Wittmann et al., 2009; Grillo et al., 2010; Muha et al., 2011).

To study this anatomy-high-performance framework we present a study of synapse loss vs. signal synchronicity and the influence on somatic calcium signals as well as simulations of large and detailed network simulations (10,000 neurons, each neuron containing 574–586 degree of freedom) of a neocortical column synthetically generated with NeuGen. In these studies we show that synapse loss, which is a major factor in neurodegenerative diseases, can be partially compensated by an increase in synaptic synchronicity, while somatic calcium signals rely strongly on the activation and frequency of action potentials. We further show that wave activation in neocortical networks is clearly driven by synapse density and that our employed simulation framework scales well on JUQUEEN, one of the high-performance computers at the German Jülich Supercomputing Center. This in turn demonstrates that large-scale network simulations do not necessarily have to come at the cost of anatomy anymore.

## 2. Materials and methods

In this section we will introduce the tools and methods used for the simulations performed in Section 3. A combination of neuron and network generating tools (Section 2.1), synapse distribution algorithms, a new approach for numerical discretization of the network topology and a parallel computing framework (Section 2.2) forms the basis of our detailed anatomical and large-scale network simulations and is integrated in a new and extendible simulation toolbox, NeuroBox (Section 2.3).

### 2.1. Generating large and anatomically detailed networks

The generation of large neural networks (containing more than 10,000 neurons) is accomplished with the neural network generator NeuGen (Eberhard et al., 2006). NeuGen uses anatomical fingerprints, i.e., experimental morphology data and standard deviations to generate anatomically consistent neurons that fit experimental mean and standard deviation. NeuGen thus generates non-identical neurons of various types—e.g., pyramidal cells and spiny stellate cells of the neocortex and hippocampus—and synaptically connects these to form neural networks. The topology of the network is described in terms of graph theory as an undirected, connected graph containing edges and vertices in three-dimensional coordinate space. NeuGen algorithms sample parameter values from experimental data distributions and incorporates two categories of synapses: *Primary* synapses representing external stimulation of the network; as well as *interconnecting* synapses which represent chemical synapses between neurons present in the network, typically formed by a presynaptic axon and a postsynaptic dendrite. The anatomy of the network can be exported to a 3D graphics format for visualization and various discrete morphology file formats that can be used in simulators such as NEURON (Hines and Carnevale, 1997) or UG 4 (Vogel et al., 2013). NeuGen is intended to provide anatomically accurate large network topologies for general purpose neuron network simulators.

**Figure 1:**
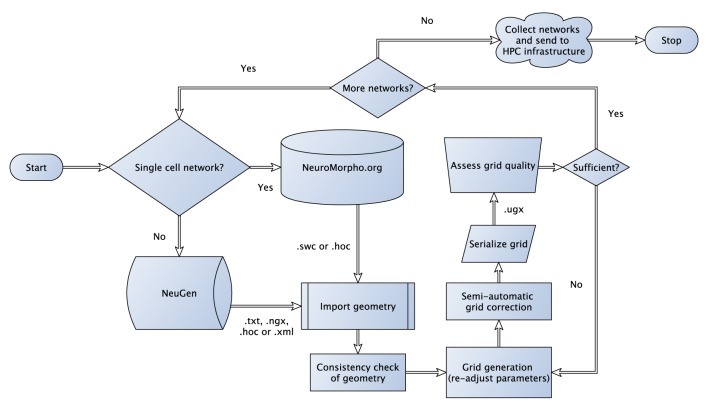
**Flowchart sketching the streamlined pipeline for the generation and subsequent transformation of neuronal network morphologies to grids suitable for large-scale network simulations**. In case of three-dimensional simulations, where one-dimensional point/line reconstructions are used to generate three-dimensional representations (see Grein et al., 2014) quality assessment of the generated grid can be performed in a semi-automatic way to allow for the best possible preparation for the subsequent numerical simulations, for instance, we check for intersecting dendrites introduced during neuron tracing.

The algorithm, which is not a growth-based algorithm, is summarized by the following steps (cf. Figure 1):

– Generate sections for each neuron based on anatomical fingerprints
– Interconnect sections of individual neurons
– Generate synapses based on a distance criterion and attach functional parameters

It is worth highlighting two parameters when discussing anatomical detail. To regulate the number of vertices for each neuron (which represents the level of detail at which neuron morphology is represented), one may adjust a parameter termed section_length, the average compartment length in μ*m*. In cases where memory consumption is a constraint, choosing an increased section length permits the creation of and simulation on larger networks (with less anatomical detail) using the same amount of memory. Secondly, the number of synapses inserted into the network may be adjusted by a global threshold parameter termed dist_synapse. If and only if the euclidean distance between two sections falls below the threshold specified by this parameter, these sections will be marked as potential synaptic contact points. Whether or not a synapse will be placed in the network depends on the type of pre- and postsynaptic neurons. A connectivity matrix specifies which classes of neurons are interconnected by synapses (Wolf et al., 2013).

**Figure 2:**
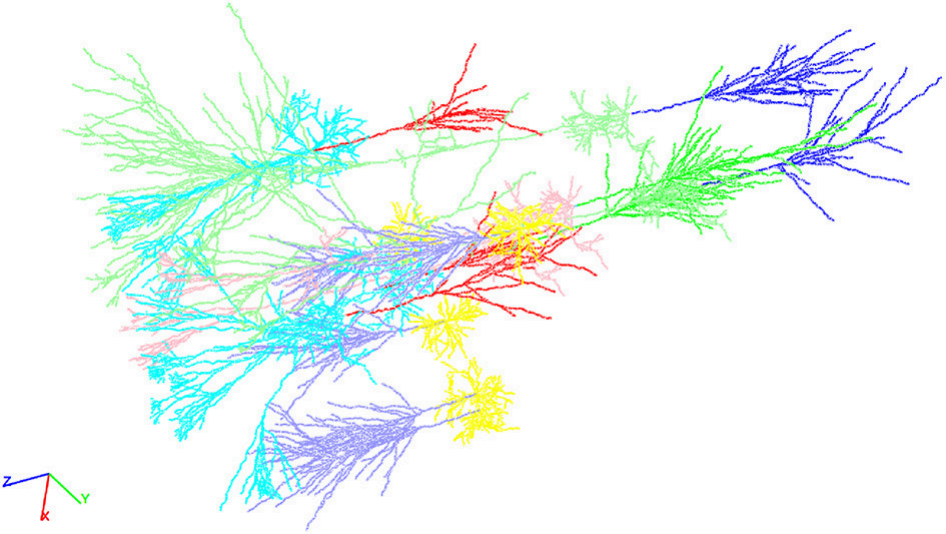
**An exemplary neocortical network consisting of a total of ten individual cells synthetically generated with NeuGen**. The cellular composition of the displayed network is identical to the networks described in Table 1 above. Visualization comprises the different cell types distinguishable by color: axons of L4 spiny stellate (lavender), axons of L2/3 pyramidal (red), axons of L5A pyramidal (bright green), axons of L5B pyramidal (blue), dendrites of L4 spiny stellate (yellow), dendrites of L2/3 pyramidal (cyan), dendrites of L5A pyramidal (rose), dendrites of L5B pyramidal (light green), somata of L4 spiny stellate (ocher), somata of L2/3 pyramidal (orange), somata of L5A pyramidal (light blue), and L5B pyramidal (brown).

Subsequent simulations need to refer to the compartments contained in the grid for simulation control setup. Therefore, an alphanumerical identifier is stored within the grid too. The identifier is a string and composed out of the cell type (e.g., pyramidal or stellate) and the compartment type (e.g., axon or dendrite) and groups all edges and vertices belonging to a given cell and compartment type (cf. Figure 2). If desired one can request the identifier to group edges and vertices based also on the section number of the compartment resulting in a fine-grained access of the network (not shown).

The network can be exported to a variety of formats including a format suitable for large neural network grid generation, e.g., a custom sparse data format based on a file format derived from TXT (plain text or compressed plain text) or a more convenient XML-based file format.

To use NeuGen in conjunction with the simulation framework UG 4 (Vogel et al., 2013, cf. Section 3), the exported morphology is exported to the UG 4 geometry format UGX (an xml-based file format). To that end, topology information of the exported network, consisting of the raw nodes and vertices, is enriched by grid attachments such as diameter information and synapses, together with their parametrizations. This procedure is implemented as a plugin for UG 4 and produces large neural networks (≥ 10, 000 neurons) in the matter of seconds (cf. Table 1).

**Table 1:** **Network creation statistics sorted by size, i.e., by number of contained cells within the network, in ascending order**.

**Vertices**	**Sections**	**Cells**	**Elapsed time [s]**	**Grid size [mb]**
1403	418	12	0.01	0.14
15535	4382	120	0.02	1.60
156596	43892	1200	0.50	16.9
1644260	465262	12000	44.7	65.6
2840213	1212108	120000	300	221.1

*The networks are composed of L5A and L5B pyramidal cells (≈ 16% each), of L4 spiny stellate cells (≈ 42%) as well as L2/3 pyramidal neurons (≈ 26%). The smallest network contains 12 and the largest network 120,000 cells in total. To create even larger networks with the same memory resources, one can decrease the number of compartments using the section_length parameter*.

In addition to directly writing UGX-files from NeuGen, it is possible to convert the following formats to UGX: SWC (commonly used in the *NeuroMorpho.org* database, Ascoli et al., 2007), HOC (widespread format utilized by NEURON, Hines and Carnevale, 1997), TXT and NeuroML. The last three file formats can be exported directly by NeuGen. NeuGen and the corresponding UG 4-plugins thus form an efficient pipeline for integrating large and anatomically realistic neural networks and publicly accessible anatomical neuron reconstructions into neuron and network simulation frameworks.

### 2.2. Simulating electrical and biochemical signals

Having established methods for generating network topologies in the previous section, we now focus on the steps from modeling electrical signals, handling membrane transport mechanisms, including synapses to discretizing the model equations by means of a new approach via finite volumes. Lastly we summarize parallel methods for efficiently solving large-scale networks.

#### 2.2.1. Model equations for membrane potential and ion species

We follow the well established cable theory (cf. Thompson, 1854; Scott, 1975) to model electrical signals on spatially resolved neuron morphologies. A neuron's morphology is given as a graph consisting of vertices in a three-dimensional space and edges connecting them. Common file formats for neuronal morphologies (such as SWC or HOC) contain radius or diameter values assigned to each vertex. We make use of this diameter in the most simplistic way, i.e., by supposing the morphology to be piecewise tubular, each piece being located around a vertex and with the radius associated to this vertex. With only very few modifications, we also implemented compartments shaped like truncated cones resulting in a continuous radius along the neurites, however, we restrict ourselves to the case of tubular compartments in the following description for the sake of simplicity. In each of the compartments, we impose the following equation expressing the membrane's role as an ideal capacitor:

(1) $$C_{m}\frac{\partial V}{\partial t}\;=\;I_{ax}+I_{m},$$

where *V* is the membrane potential, *C*_*m*_ is the capacitance of the compartment and *I*_*ax*_, *I*_*m*_ are the axial and transmembrane (inward) electric currents, respectively. The compartment's capacitance *C*_*m*_ depends on its shape and can be expressed in terms of a membrane-specific constant *c*_*m*_,

$$C_{m}=c_{m}\cdot 2\pi al,$$

where *a* and *l* are the radius and the axial length of the compartment, respectively.

**Figure 3:**
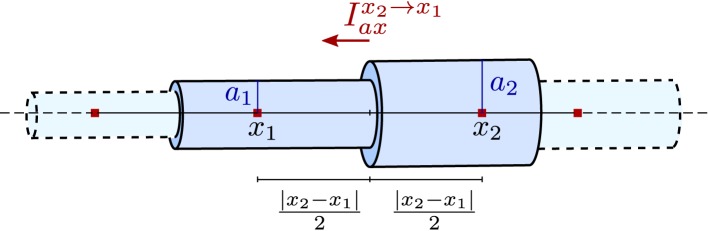
**Illustration of the piece-wise tubular compartments of the Finite Volume cable equation model and the definition of axial ohmic flux**.

Axial currents need to be calculated at both ends of a compartment, at the interface with the neighboring compartments. They are assumed to be purely ohmic in nature and are expressed in terms of voltage between the two vertices associated with the neighboring compartments:

$$I_{ax}^{x_{2}\to x_{1}}=\frac{V\text{​}\left(x_{2}\right)-V\text{​}\left(x_{1}\right)}{\frac{r_{c}}{\pi }\int_{x_{1}}^{x_{2}}{\left(a\left(x\right)\right)}^{-2}\;dx}=\frac{V\text{​}\left(x_{2}\right)-V\text{​}\left(x_{1}\right)}{\frac{r_{c}}{2\pi }\left(a_{1}^{-2}+a_{2}^{-2}\right)\left|x_{2}-x_{1}\right|},$$

where *r*_*c*_ is a material constant, the specific resistance of the cytosol, *x* is the axial coordinate, and *x*_1_, *x*_2_ as well as *a*_1_, *a*_2_ are depicted in Figure 3. Note that the former equation implicitly assumes that the extracellular potential is constant in space.

Finally, the transmembrane current *I*_*m*_ into the compartment is expressed in terms of electrical flux density *i*_*m*_ as

(2) $$I_{m}=i_{m}\;\cdot \;2\pi al$$

and depends on transport mechanisms (e.g., Hodgkin-Huxley-type channels, Na/K pumps, leakage), synapses and electrodes definable on the membrane.

In order to track individual ion species, concentrations for K^+^, Na^+^, and Ca^2+^ or any other ion type can be added to the model. Each of the species satisfies a diffusion-convection equation in axial direction and is coupled to transport mechanisms in the plasma membrane.

Note that as these ions are charged, they are affected by potential gradients in reality—and conversely, for the same reason, their concentrations directly affect the potential. A physically more accurate model of ionic movement in neurons incorporating both electric and diffusive properties of individual ion species is electro-diffusion. It has ben demonstrated that the modeling error introduced by using the cable equation can be prominent in thin compartments (Qian and Sejnowski, 1989) or where three-dimensional structural detail is concerned (Lopreore et al., 2008).

#### 2.2.2. Membrane transport mechanisms

What is truly at the heart of most neuronal simulations is transport across membranes. We have defined an interface allowing the addition of arbitrary transport mechanisms to the electrical model in the transmembrane current density term *i*_*m*_ of Equation (**2**). These transport mechanisms are granted access to the underlying grid as well as to the unknowns of the voltage and ion species equations. Thus, they are able to declare and calculate their own sets of states, which may depend on given ones and vary in space and in time—like the gating parameters *m*, *n* and *h* in classical Hodgkin-Huxley-type channels governed by ordinary differential equations in time which depend on the membrane potential (Hodgkin and Huxley, 1952). As the dependence of inner states of membrane transport systems on the potential and on ion concentrations is typically strongly non-linear, we have decided (in the interest of fast computation) to include transmembrane currents only by an explicit scheme, i.e., inner states are updated before any time step of the solution process using only the solution from the previous time step.

The concept is not unlike the NMODL model description language for NEURON by Hines and Carnevale (1997, 2000). In fact, we have developed an automated translation unit that can convert existing NMODL files to C++ source code compilable in our framework.

#### 2.2.3. Synapses

Glutamate being the primary excitatory neurotransmitter in most synapses of the central nervous system, we define excitatory synaptic input localized at dendrites as the postsynaptic response of AMPA or NMDA receptors to presynaptic glutamate signals. AMPA and NMDA receptors, cation channels that become permeable in glutamate-bound state and thereby exhibit a conductance change in direct response to incoming presynaptic spikes, induce transmembrane flux of sodium, potassium and calcium ions causing a local excitatory depolarization of the membrane potential.

In our simulations we distinguish two general categories of synapses: *Primary* synapses connected to dendrites as the postsynaptic side,—they are used to initialize activity in single cells as well as networks and represent connections to other neurons not included in the simulation. The second category are synapses connecting dendrites and axons both present within a network morphology. We call these *interconnecting* synapses.

As there is no information on the presynaptic side of primary synapses, the common and simple approach of *alpha functions* provides a reasonable approximation to model postsynaptic conductance profiles (Roth and van Rossum, 2009):

(3) $$g(t)=g_{\text{max}}\frac{t-t_{\text{onset}}}{\tau }\exp\left(-\frac{t-t_{\text{onset}}-\tau }{\tau }\right),$$

where *g*_max_ denotes the maximal conductance, τ the rise/decay and *t*_onset_ the arrival time of a single presynaptic spike. Note that *g*_max_ occurs at *t* = *t*_onset_+τ. The synaptic current *I*_ps_(*t*) is then defined by

(4) $$\begin{array}{ll} I_{\text{ps}}(t) & =g(t)(V(t)-E_{\text{rev}}), \end{array}$$

with *g*(*t*) given by (3) for *t*_onset_ ≤ *t* ≤ *t*_onset_ + 6τ and *g*(*t*) = 0 otherwise. *V*(*t*) denotes the current postsynaptic membrane potential and *E*_rev_ a reversal potential. For glutamatergic synapses, we use *E*_rev_ ≈ 0 mV (Purves et al., 2001).

Interconnecting synapses are activated upon rise of the presynaptic membrane potential above a threshold *V*_th_ and the following current *I*_is_(*t*) to the postsynaptic end is modeled according to a bi-exponential activity function:

(5) $$t_{\text{max}}=\;\frac{\tau_{1}\tau_{2}}{\tau_{2}-\tau_{1}}\log\left(\frac{\tau_{2}}{\tau_{1}}\right),$$

(6) $$n\;=\;{\left(\exp\left(-\frac{t_{\text{max}}}{\tau_{2}}\right)-\exp\left(-\frac{t_{\text{max}}}{\tau_{1}}\right)\right)}^{-1},$$

(7) $$I_{\text{is}}(t)\;=\;g_{\text{max}}\left(V-E_{\text{rev}}\right)\;n\left(\exp\left(-\frac{t}{\tau_{2}}\right)-\exp\left(-\frac{t}{\tau_{1}}\right)\right),$$

where *g*_max_ is the maximal conductance; *E*_rev_ is a reversal potential; τ_1_ and τ_2_ are constants regulating rise and decay time of the conductance; *t*_max_ designates the point in time (after initial activation) at which the conductance is maximal, and the factor *n* normalizes the conductance such that its value is *g*_max_ at *t*_max_.

Synaptic currents—like all other trans-membrane currents—are evaluated using the solution for the potential of the previous time step only. This has significant benefits in parallel computation, as there is no direct coupling of solutions for the next time step between cells connected to one another by synapses.

#### 2.2.4. Activation patterns of primary synapses

Our implementation provides a method to set generic activation patterns for a given set of input synapses in the computational domain. To achieve that, we introduce the continuous random variables *X*_onset_ and *X*_τ_ for the timing parameters *t*_onset_ and τ [cf. Equation (3)], respectively. Both of which we assume to be normally distributed, i.e., *X*_onset_ ~ N ($\mu_{\text{onset}},\sigma_{\text{onset}}^{2}$) and *X*_τ_ ~ N ($\mu_{\tau },\sigma_{\tau }^{2}$) with probability density functions given by:

(8) $$\begin{array}{l} f_{N}(\;x_{\xi },\mu_{\xi },\sigma_{\xi }^{2})=\frac{1}{\sigma_{\xi }\sqrt{2\pi }}e^{-\frac{1}{2}{\left(\frac{x_{\xi }-\mu_{\xi }}{\sigma_{\xi }}\right)}^{2}},\;\xi \in \{\text{onset},\;\tau \} \end{array}$$

After specification of a peak conductance *g*_max_, a mean onset time μ_onset_ and duration μ_τ_ of synaptic activity as well as corresponding standard deviation values σ_onset_ and σ_τ_, the parameters *t*_onset_ and τ are set to random values drawn from the above normal distributions $N\;\left(\mu_{\text{onset}},\sigma_{\text{onset}}^{2}\right)$ and $N\;\left(\mu_{\tau },\sigma_{\tau }^{2}\right)$, respectively.

#### 2.2.5. Spatial distribution of primary synapses

Given neuron morphologies (defined as graphs in three-dimensional coordinate space), we attach all information parameterizing synapses to the dendritic edges they are associated to. The distribution is managed by the C++ class SynapseDistributor. It provides methods to create new or delete existing ones to user-specified statistical distributions.

In our studies, we assume a uniform distribution of *n*_syn_ ∈ ℕ synapses on the edge sample space

(9) $$\begin{array}{l} S_{\text{edge}}:=\{e_{i}\;|\;i=1,...,\;n_{\text{edge}}\} \end{array}$$

of the basal and apical dendrites. For this purpose, we consider a discrete random variable $X_{\text{syn}}^{i}$, *i*∈{1, …, *n*_syn_}, for the *i*-th synapse to be distributed. With every draw $X_{\text{syn}}^{i}$ can thereby take one of the edge indices *j*∈{1, …, *n*_edge_} as value, i.e., $X_{\text{syn}}^{i}=x_{j}^{i}:=j$. To account for the heterogenous edge lengths every edge index is assigned an associated probability given by the following *probability mass function*:

(10) $$P\;(X_{\text{syn}}^{i}=x_{j}^{i})=p_{j}^{i}\;:=\frac{\|e_{j}{\|}_{2}}{\sum_{k=1}^{n_{\text{edge}}}\|e_{k}{\|}_{2}}$$

The exact location of the *i*-th synapse $x_{j}^{i}$ on the *j*-th edge is then drawn from a continuous uniform distribution in the range (0, 1).

#### 2.2.6. Discretization and solution

We use a first-order (vertex-centered) Finite Volume (FV) scheme. This type of discretization method is well-suited for any type of problem resulting from a conservation law. In a FV scheme, one typically has a conservation formulation like the following:

(11) $$\frac{\partial \rho }{\partial t}\;=\;-\text{div }\vec{j}\text{ on the domain }\Omega ,$$

where ρ is the density of a conserved quantity, $\vec{j}$ is a flux density of the same quantity. In our case, ρ represents the charge density for the voltage equation and the ionic concentration for the species equations; the flux densities are given by the electric current density and the ionic flux density, respectively. The conservation equation is then transformed into a system of ordinary—i.e., non-differential—equations by partitioning the domain on which the equation holds into so-called control volumes (in our case, those are exactly the compartments as defined above),

$$\Omega =\underset{i}{\cup }\Omega_{i},$$

then by integrating Equation (11) on each control volume (thus ensuring local conservation)

$$\int_{\Omega_{i}}\frac{\partial \rho }{\partial t}\;dx\;=\;\int_{\Omega_{i}}-\text{div }\vec{j}\;dx\;\left(=\;-\int_{\partial \Omega_{i}}\vec{j}\cdot {\vec{n}}_{i}\;dS\right)\;\forall i,$$

and finally by assuming the unknown function to be part of some finite-dimensional space (in our case: piecewise linear) in order to be able to represent it by a finite number of unknowns which can be used to express the integrals explicitly in a system of ordinary equations,

$$\rho =\sum_{k}\lambda_{k}\rho_{k},$$

where {ρ_*k*_} are a known basis of the finite-dimensional function space; while {λ_*k*_} are the coefficients in the corresponding representation of ρ and, at the same time, the unknowns of the resulting system of equations.

Time discretization is achieved by an Euler scheme, backwards with respect to axial fluxes and forward with respect to radial fluxes. The latter treatment results in a step size requirement for the time integration, the numerically well-known Courant-Friedrichs-Lewy (CFL) condition (Courant et al., 1928). In this particular case this condition states: The more trans-membrane flux there is, the smaller the time step has to be chosen. If the requirement is not met (i.e., if the time step size is chosen too big) the solution will “explode,” meaning that it will tend to infinity very rapidly. In order to prevent such instability, we calculate and use an estimate for the allowed step size. Thus, our time step is neither too big (“explosion”) nor too small (inefficiency).

Discretization is performed using the numerical framework UG 4 (Vogel et al., 2013). It is written in C++ and simulations can be set up and run using the widespread scripting language Lua, which makes this framework easy to use without learning a highly specialized language of its own.

Solution of the symmetric system of linear equations emerging from the discretization is also done within the UG 4 framework. The tree structure of neurons allows for an efficient usage (i.e., with linear runtime complexity in terms of the number of unknowns) of a direct solver if the unknowns are numbered in such a way that, in each line of the matrix, there is at most one non-zero entry to the right of the diagonal. We use a Cuthill-McKee (Cuthill and McKee, 1969) ordering to guarantee this. We solve by calculating the LU decomposition in a sparse matrix format.

#### 2.2.7. Parallelization

As UG 4 comes with full MPI support for parallel calculations, the inevitable usage of large-scale computer facilities for the simulation of large networks is straight forward. Partitioning of the domain can be performed using Metis 5.0 (Karypis and Kumar, 1998) and can be achieved on two levels:

In large networks, whole neurons can be assigned to the processors (as described for Neuron in Migliore et al., 2006), resulting in an “embarrassing” parallelism, since there is no direct coupling between the neurons if synaptic events triggered on the presynaptic side in one time step are taken into account only in the next time step on the postsynaptic side. If whole neurons can be distributed in such a way that the processors' workloads are well balanced, this will be the preferred way of parallelizing, as the solution of the problem works exactly like in the serial case and communication is only needed at active synapses.

On a second parallelization level, it is also possible to cut neurons and assign their parts to different processors. The process of solving the system of equations is a little bit more involved then. Assuming the system to be solved on a processor is

Ax=b,

then the iterative solving process on each processor is defined by the following pseudo-code:

 *x*_0_ = solution from the precedent time step

 *d* = *d*_0_ = *b*−*Ax*_0_ (“defect” vector)

 **while** |*d*| < |*d*_0_| · reductionFactor on any processor **do**

    *c* = *A*^−1^*d* (calculate correction)

    Sum up (over all processors) the corrections in all cutting points and store back in c.

    *x* = *x*+*c* (update solution)

    *d* = *b*−*Ax* (update defect)

 **end while**

In order for this to work, the process-wise matrices *A* need to be stored “additively,” i.e., the entries of the global system matrix must be equal to the sum of the corresponding entries in the process-wise matrices (where existent).

It usually takes about five to fifteen iterations until convergence is achieved, depending on how many neurons are cut and at which locations. Of course, in the case where no neuron is cut by the distribution of the network, the iteration will converge in one step. The gain in computation time from parallelizing on this level is not as big as from distributing whole neurons, obviously—however, it can still provide some speedup as it is not *solving* the system which takes the most time, but *setting it up* in the first place.

### 2.3. Simulation workflow

The efficiency of simulating large and complex systems in neuroscience strongly depends on the scaling properties of code on high performance computers (Section 2.2.7). Additional aspects when looking at efficiency are the time invested for setting up a model, the computational tools, compiling and visualizing data and finally accessibility to an extendible code basis.

The simulation toolbox NeuroBox focusses on these aspects by allowing users to compile visual or script-based workflows. Workflows can define models, numerical tools and include third-party tools, such as ImageJ (Schneider et al., 2012). The multi-level design, founded on the multi-physics engine UG 4 (Vogel et al., 2013) and the Visual Reflection Library (VRL, Hoffer et al., 2013), allows non-experts intuitive access to advanced numerical methods for solving anatomically detailed biophysical models. NeuroBox is an open-source project hosted on github and thus is conceived as a modular and extendible C++ framework, where new biological components such as ion channels, receptors, synapse types etc. can be added manually or through an NMODL importer. This section briefly introduces script-based and visual workflow design and examples of the extendibility of NeuroBox, which as a platform is capable of hosting large multi-domain workflows.

#### 2.3.1. Using lua scripts

**Figure 4:**
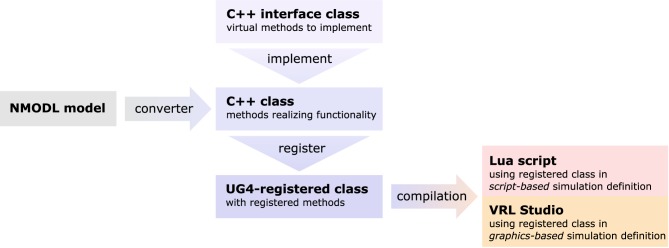
**Sketch of the NeuroBox framework, using an ion channel model as an example**. Functionality of the channel is implemented in a C++ class deriving from a pre-defined interface. The implementation can be automatically generated by conversion from NMODL model file. Registering the class at the UG 4 registry and compiling makes the channel available for usage in simulations defined either by a Lua script or by a graphical workflow representation using VRL-Studio.

**Figure 5:**
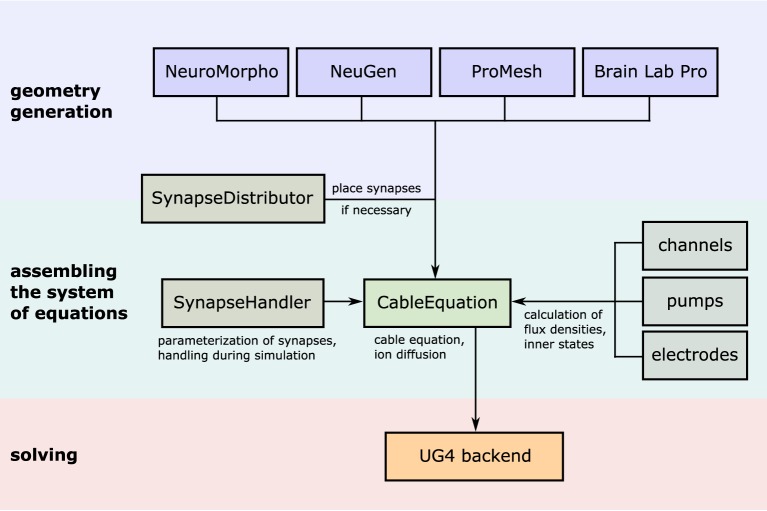
**Illustration of the simulation workflow**. After creation of a neuronal (network) morphology, the system of linear equations emerging from the cable equation is assembled by the central class CableEquation; synapse handling (i.e., activation, calculation of fluxes, parallel coordination) is taken care of by the class SynapseHandler, while all trans-membrane fluxes are handled by individual classes which all derive from a common interface known to the CableEquation class. The system is solved using UG4 solvers and parallelism.

The complete process of setting up and solving a problem in parallel is handled internally by UG 4. In order to use its functionality, we developed our code as a UG 4 plugin and compile against the UG 4 libraries. We register our classes and functions at the UG 4 registry (this is done in the C++ code) in order to make them available at the Lua script level, where a simulation can then be formulated using the registered functionality (in addition to any valid Lua command; see Figure 4). A schematic representation of what a typical simulation workflow looks like is shown in Figure 5, an example script with extensive comments is provided in Listing 1 in Supplementary Material.

#### 2.3.2. Using graphical workflows

We take advantage of the open source software VRL-Studio (Hoffer et al., 2013) to represent simulation workflows graphically. Each class and function registered in the UG 4 registry can be represented in VRL-Studio. This allows any user to put together a simulation by dragging and dropping the graphical representations of involved objects (like instances of the cable equation discretization, the channel and pump mechanisms or the synapse handler) and adding application of their methods with only a few clicks. Scripts are not necessary but possible. The important aspect is that VRL-Studio can combine textual and visual programming in a single interactive development environment. For some aspects, script-based development has many advantages. Therefore, VRL-Studio provides access to the UG 4 APIs. Lua-scripts can be integrated into the visual workflow. A Lua editor with advanced autocompletion support allows for intuitive Lua-based development. Even more important is the fact that VRL-Studio workflows can integrate any Java library, such as ImageJ and JFreeChart. Automatic GUI generation works for these external libraries as well. Users can easily extend existing workflows with custom Groovy scripts, e.g., for pre- and post-processing. Custom scripts are also available as graphical components. Using external libraries in custom scripts is a powerful tool for adding domain-specific knowledge to the NeuroBox platform.

**Figure 6:**
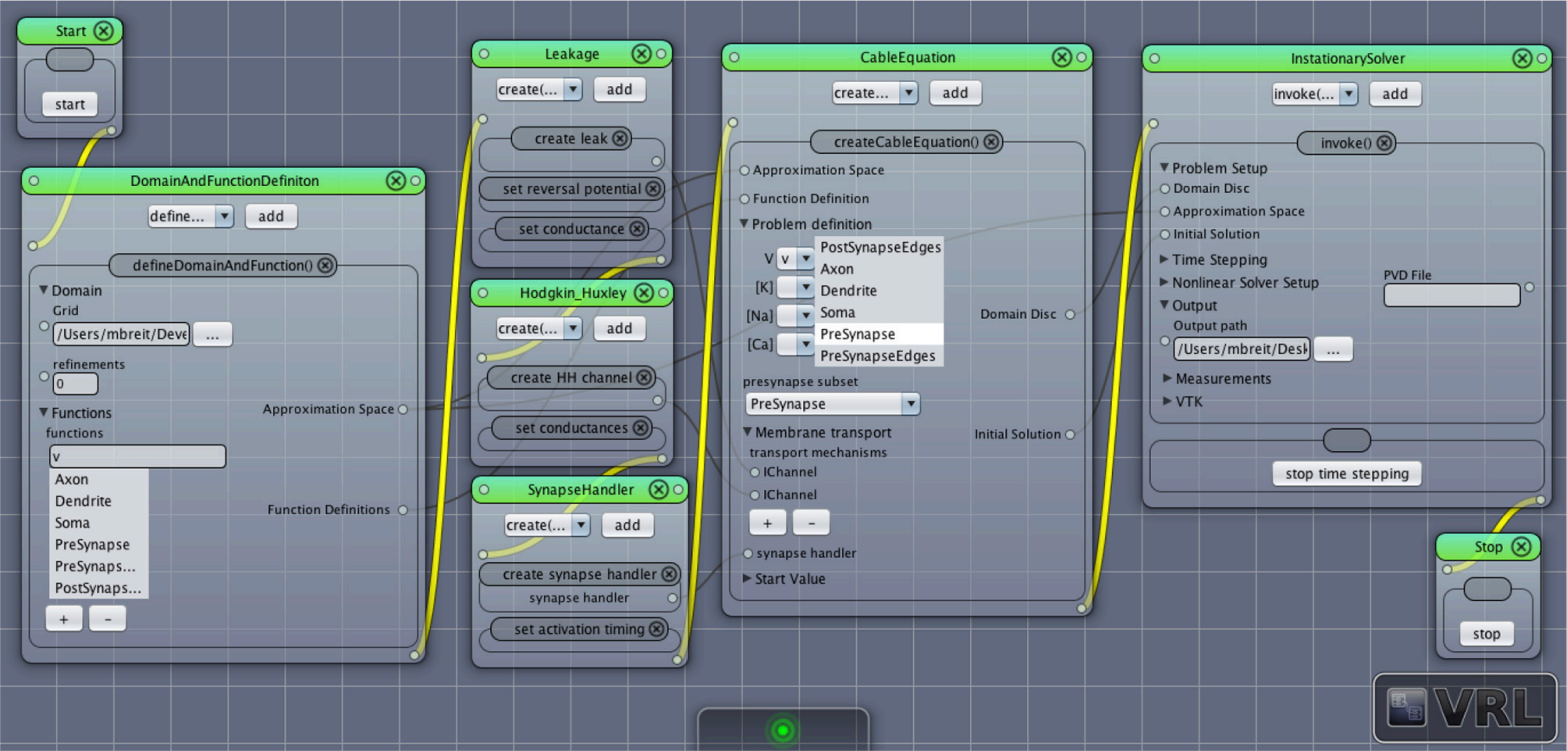
**Screenshot of a simple network simulation workflow assembled in VRL-Studio**. Each window represents an object, the named panels in the window represent a method call with the contents of the panel as parameters. The control flow is defined by the yellow connections, data transfer between objects is marked by gray connections.

Typically the following steps can be followed to set up a new NeuroBox workflow (a screenshot of a simple graphical simulation workflow created in this way is depicted in Figure 6):

1. The first step is the definition of the computational domain (the neuronal morphology) and the unknown functions (membrane potential, ion concentrations) to be computed. This is done by adding an instance of DomainAndFunctionDefinition to the canvas and selecting the grid file as well as names for the unknown functions and subsets of the domain they are supposed to be defined on (subsets defined in the geometry file can be chosen from a list).
2. The following step in the workflow is the definition of all membrane transport mechanisms (channels and pumps) as well as a synapse handler (if any synapses are present in the domain). All of them may be drag-and-dropped onto the canvas and then parameterized as needed.
3. An instance of the central CableEquation class is added to the canvas. All defined membrane transport mechanisms as well as the synapse handler (if applicable) need to be connected by (gray) data connections. Initial conditions need to be supplied.
4. A solver is added to the canvas. The time stepping parameters need to be set as required. Output options can be specified.
5. Workflow connections (yellow) are drawn establishing the unique order in which the objects are created and their methods called. Any object receiving data input from another object needs to be behind that object in the workflow, i.e., the objects need to be in the order indicated by the enumeration here.
6. The simulation may be started, output can be observed in the log window, recorded and visualized.

#### 2.3.3. Adding functionality

As there is an abundance of membrane transport mechanisms and even more models trying to describe them, it is hardly possible to implement all of them in advance. In order to support a large pool of available models, we wrote a file converter that will produce C++ code suitable to be compiled with our UG 4 implementation from any model file conforming to Neuron's NMODL description language (Hines and Carnevale, 2000). Of course, membrane transport models can also be implemented directly on the C++ level, implementing the required methods of a pre-defined interface class. This requires writing code for the initialization and updating (typically: evolving some kind of gating variables, expressed in terms of ordinary differential equations) of a model as well as code for the computation of the ion and charge flux through the membrane effectuated by the model. After registration of a new model at the UG 4 registry and compilation of the corresponding code, the model can be used on the Lua script level or on the graphical workflow level in VRL-Studio. The whole process is depicted schematically in Figure 4.

### 2.4. Setups for our simulations

#### 2.4.1. Synapse loss simulations

We conducted *in silico* experiments investigating the impact of synapse loss in various activation patterns, particularly focussing on the effects it has on the formation of action potentials and the somatic calcium signal. For the simulations we chose a layer 3 pyramidal cell from the rat neocortex reconstructed by Radman et al. (2009), which was well suited to serve as reference cell for further studies as its reconstruction comprised the complete description of soma, dendrites and axon. The corresponding neuronal morphology is publicly available in the SWC file format as part of the *NeuroMorpho.org* database (Ascoli et al., 2007) under the name 13-L3pyr-77. It was converted to the UGX file format to meet UG 4 format specifications.

Subject to the discrete probability distribution specified in section 2.2.5, *N* = 100 distributions of *n*_syn_ = 1000 synapses each were drawn from the sample space *S*_edge_ defined in (12). We simulated synapse loss by successively removing portions of the previously created synapses uniformly from the neuron.

Regarding synapse activity we used a maximal conductance of *g*_max_ = 1.2 nS and a constant rise/decay time of τ = 0.4 ms representing a fast AMPA receptor channel parameterization (Gabbiani et al., 1994) throughout the simulations. We compared three levels of input pattern synchrony, namely: complete synchrony (σ_onset_ = 0), moderate asynchrony (σ_onset_ = 5 ms), and high asynchrony (σ_onset_ = 10 ms).

A fraction of 0.2–4% of the current through AMPA receptor channels is carried by calcium ions, depending mainly on the exact AMPA subtype (Burnashev et al., 1995; Garaschuk et al., 1996). As we did not consider calcium buffer (calmodulin, calbindin) reactions in our simulations, we reduced this amount to 0.1% in order to (roughly) represent fast binding of free calcium to these buffers. Calcium dynamics were also regulated by N-type voltage-dependent calcium channels modeled according to Borg-Graham (1999) and NCX and PMCA pump mechanisms (first-order, second-order Hill-type model, resp.). A leakage term was added to ensure zero-flux for the equilibrium state.

#### 2.4.2. Network simulations

For the simulations in Section 3.2.2, we used NeuGen to create five neocortex geometries composed of 3500 L2/3 pyramidal; 3500 L4 spiny stellate; 1500 L5A and L5B pyramidal cells each whose somata were contained in a box with extensions of about 0.5 mm × 0.5 mm × 1 mm (length × width × depth), resulting in a cell density which is of the same order of magnitude as reported by Rockel et al. (1980). In each of the five geometries, NeuGen distributed an average of 30 primary synapses per L4 spiny stellate cell and an average of 25 per L5B pyramidal (cf. Constantinople and Bruno, 2013) for thalamic input. Interconnecting synapses were created wherever axon and dendrite from compatible neuron types came close enough, with the critical distance dist_synapse (cf. Section 2.1) being 1 µm for the first network, 2 µm for the second and so on. The numbers of synapses thus created show a cubical dependence on the critical creation distance (cf. Table 2), which is to be expected, as the sphere around any dendritic point within which axonal points eligible for connection through a synapse are located grows cubically in volume with increasing radius.

**Table 2:** **Number of synapses connecting specific cell types for various critical creation distances dist_synapse (averaged w.r.t. postsynaptic cell type) in the 10,000 cell networks created by NeuGen**.

**Synapse creation distance [μm]**	**1**	**2**	**3**	**4**	**5**
L4 → L2/3	7.5	59	200	474	926
L4 → L5A	2.7	22	74	173	338
L2/3 → L5A	1.4	11	39	92	179
L2/3 → L5B	1.2	9.5	33	77	150
L2/3 → L2/3	0.48	3.8	13	30	58

Axonal, dendritic and somatic membranes contained classical Hodgkin-Huxley-type sodium and potassium channels. Their flux density is described by

(12) $$i_{hh}\;=\;c(T)\left(g_{K}n^{4}(V-E_{K})+g_{Na}m^{3}h\;(\;V-E_{Na})\right),$$

(13) $$\frac{\partial n}{\partial t}=\;c(T)\left(\alpha_{n}\left(V\right)\left(1-n\right)-\beta_{n}\left(V\right)n\right),$$

(14) $$\frac{\partial m}{\partial t}=\;c(T)\left(\alpha_{m}\left(V\right)\left(1-m\right)-\beta_{m}\left(V\right)m\right),$$

(15) $$\frac{\partial h}{\partial t}=\;c(T)\left(\alpha_{h}\left(V\right)\left(1-h\right)-\beta_{h}\left(V\right)h\right),$$

where *c*(*T*) is a temperature-dependent constant with a value of about 3.2 at 37 °C (roughly taken from Collins and Rojas, 1982; Tiwari and Sikdar, 1999); *g*_*K*_, *g*_Na_ are (location-specific) conductance constants; *E*_*K*_ and *E*_Na_ Nernst potentials; and the rate functions α and β are taken from the original Hodgkin and Huxley publication (Hodgkin and Huxley, 1952).

We used a leakage flux density to achieve zero net flux at resting potential:

(16) $$\begin{array}{ll} i_{l}\; & =\;c(T)\;g_{l}\left(V-E_{l}\right), \end{array}$$

where *g*_*l*_ is the leakage flux conductance and *E*_*l*_ an (artificial) reversal potential calibrated to ensure zero membrane net flux at resting potential.

For initialization, the membrane potential was set to the resting potential of -0.065 V globally, voltage-dependent potassium and sodium channels were also set to their resting states. At the beginning of the simulation, thalamic input synapses were activated using an alpha function [cf. Equation (3)] with *t*_onset_, τ drawn from normal distributions with (μ_*onset*_, σ_*onset*_) = (5 ms, 2.5 ms), (μ_τ_, σ_τ_) = (2.5 ms, 0.1 ms), respectively, and *g*_max_ = 1.2 nS.

Synapses between cells of the network were exclusively excitatory glutamatergic in nature and modeled as described in Section 2.2.3 using a parameterization which represents a fast AMPA receptor channel (Gabbiani et al., 1994). The maximal conductance parameter of synapse *S* with a presynaptic neuron of type *T*_1_ and a postsynaptic neuron of type *T*_2_ is calculated by NeuGen according to the formula

(17) $$\begin{array}{ll} g_{\text{max}}\left(S\right) & =\;\left(1+0.001\;\cdot \text{ dsd​}\left(S\right)\right)\;\cdot \;g_{s}\left(T_{1},T_{2}\right), \end{array}$$

with dsd(*S*) being the post-synapse's distance to the soma in µm; and a type-specific base conductance the values of which are summed up in Table 3.

**Table 3:** **Base synaptic conductance values for connections between different types in units of nS**.

**Synaptic type: pre- \post-**	**L2/3 pyr**.	**L4 stell**.	**L5A pyr**.	**L5B pyr**.
L2/3 pyr.	1.0	–	0.8	0.3
L4 stell.	0.7	1.6	0.6	–
L5A pyr.	0.5	–	2.0	–
L5B pyr.	–	–	–	1.3

*Connections that are not created by NeuGen are marked by a dash*.

All other synaptic parameters were the same for each synapse. No delay through neuro-transmitter release and diffusion was considered. All parameter values for the network simulations are summed up in Table 4.

**Table 4:** **Parameters for the large-scale network simulation**.

**Parameter**	**Meaning**	**Value**	**Unit**
*r*_*c*_	Specific resistance of the cytosol	1.5	Ω m
*c*_*m*_	Specific capacitance of the membrane	1 × 10^−2^	F m^−2^
$g_{K}^{a}$	Specific potassium channel conductance of the axonal membrane	4 × 10^2^	S m^−2^
$g_{K}^{s}$	Specific potassium channel conductance of the somatic membrane	2 × 10^2^	S m^−2^
$g_{K}^{d}$	Specific potassium channel conductance of the dendritic membrane	3 × 10^1^	S m^−2^
$g_{\text{Na}}^{a}$	Specific sodium channel conductance of the axonal membrane	3 × 10^4^	S m^−2^
$g_{\text{Na}}^{s}$	Specific sodium channel conductance of the somatic membrane	1.5 × 10^3^	S m^−2^
$g_{\text{Na}}^{d}$	Specific sodium channel conductance of the dendritic membrane	4 × 10^1^	S m^−2^
$g_{l}^{a}$	Specific leak conductance of the axonal membrane	2 × 10^2^	S m^−2^
$g_{l}^{s}$	Specific leak conductance of the somatic membrane	1	S m^−2^
$g_{l}^{d}$	Specific leak conductance of the dendritic membrane	1	S m^−2^
*E*_*K*_	Potassium Nernst potential	−0.09	V
*E*_Na_	Sodium Nernst potential	0.06	V
$E_{l}^{a}$	Leak reversal potential of the axonal membrane	−0.066148458	V
$E_{l}^{s}$	Leak reversal potential of the somatic membrane	−0.030654022	V
$E_{l}^{d}$	Leak reversal potential of the dendritic membrane	−0.057803624	V
*V*_*r*_	Resting potential (global)	−0.065	V
*c*(*T*)	Temperature factor for HH channel activity	3.21	
τ_1_	Time constant in interconnecting synapses	2 × 10^−4^	s
τ_2_	Time constant in interconnecting synapses	1.7 × 10^−3^	s
*E*_rev_	Reversal potential for interconnecting synapse influx	0	V
*V*_th_	Threshold potential for interconnecting synapse activation	−0.01	V

Simulations were performed on 160 processors for a simulated time period of 20 ms and took about two hours. Parallel scaling results for this type of problem are presented in Section 3.2.1.

## 3. Results

### 3.1. Influence of synapse loss on formation of action potentials and somatic calcium signal

The human brain is one of the most complex structures known in the universe. It consists of nearly 100 billion nerve cells, each of which is entangled in a dense and constantly adapting network of massive information exchange. On average, a single neuron is linked with 10,000 to 100,000 other neuronal or non-neuronal cells via synapses (Cragg, 1975). Brain function relies essentially on those highly dynamic synaptic connections.

In this part of our study, we investigate the three-dimensional spatial distribution and activity pattern in time of glutamatergic synapses in neurons of the cerebral cortex. Both are key factors to the integrative properties of the cell. For this purpose, we have developed a tool for automatic placement of synaptic functionality onto neuron morphologies. We apply this tool to systematically assess the impact of activation patterns on the signal processing in single neurons. In particular, we perform *in silico* experiments where we successively knock out synapses at dendritic locations. We thus investigate situations where synapse loss contributes to pathological states e.g., Alzheimer's disease (Scheff et al., 2006). At the same time, we address the question under which circumstances the neuron will sustain its integrative capability. More precisely, how does impulse conductance and especially the initiation of action potentials at the axon hillock depend on the number of input synapses and their signal synchrony? Does a higher input signal synchrony sustain action potential initiation during increasing synapse loss? The degree of synchrony is defined by the size of the standard deviation from a given mean value. In our experiments, we vary the standard deviation of the start time σ_onset_ of synaptic excitations.

In the following sections, we present the results of a series of *in silico* experiments on a layer 3 pyramidal cell from the rat neocortex (cf. Section 2.4.1), in which we compare three levels of input pattern synchrony, namely: complete synchrony (σ_onset_ = 0), moderate asynchrony (σ_onset_ = 5 ms), and high asynchrony (σ_onset_ = 10 ms). We randomly distributed 1000 excitatory synapses on the geometry in 100 sample configurations. In each of these 100 configurations, we gradually increased synapse loss and analyzed the neuron's capability of creating action potentials, and at the same time, recorded corresponding calcium levels within the soma.

#### 3.1.1. Generation of action potentials

**Figure 7:**
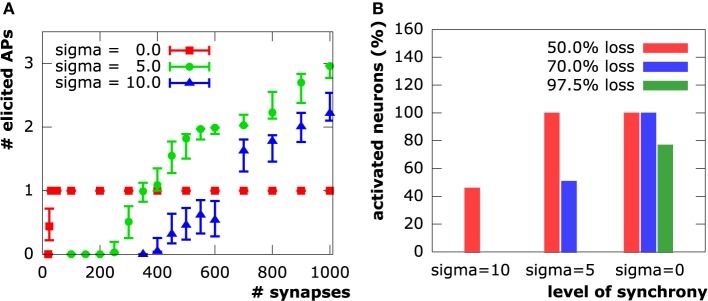
**(A)** Number of evoked action potentials in simulations of a layer 3 pyramidal neuron upon synaptic activation at varying levels of synapse loss. Experiments on *N* = 100 sample distributions of synapses with initiation of synaptic activity drawn from a normal distribution $N\left(3\sigma_{\text{onset}},\sigma_{\text{onset}}^{2}\right)$ in units of ms. Square, circle and triangle symbols represent mean values, vertical bars show standard deviations. **(B)** Synapse input synchrony counteracts synapse loss. The y-axis indicates the percentage of simulated cells that evoked at least one action potential upon synaptic activity in the three examined levels of synchrony (σ_onset_∈{45ms, 15ms, 0ms}) at three different levels of synapse loss.

Both moderate (μ_onset_ = 15 ms, σ_onset_ = 5 ms) and high (μ_onset_ = 30 ms, σ_onset_ = 10 ms) asynchrony cases show a strong action potential spike train response to the initial synapse distribution. The number of spikes ranges from two to three in the moderate asynchrony case and from one to three in the high asynchrony case (Figure 7). The synchronous setup, however, produced exactly one action potential for the initial distribution of 1000 synapses in all samples. Only the cation influx at new synapses perpetually being active in the asynchronous cases can induce the repetitive spiking, while cation influx through all synapses is completely compensated by potassium efflux during hyper-polarization in the synchronous case. The number of action potentials decreased with increasing synapse loss in both asynchronous cases until complete signal breakdown (in at least 90 % of the sample patterns) at 75% synapse loss in the moderately and at 60% in the highly asynchronous case. In contrast, synchronous activation patterns sustained generation of an action potential up to a loss of about 97.7% (corresponding to 23 synapses).

#### 3.1.2. Calcium signaling

**Figure 8:**
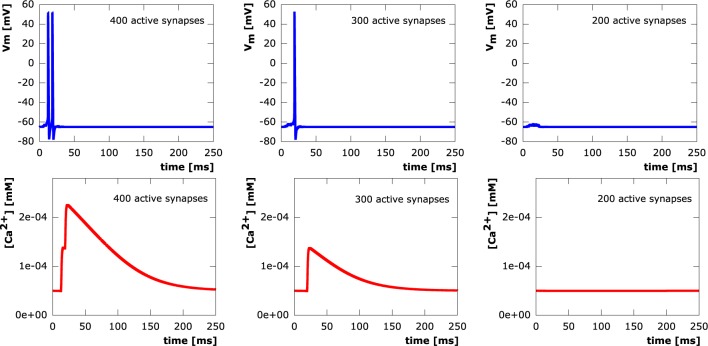
**Courses of the membrane potential in mV (row 1) and calcium concentrations in mM (row 2) measured at the soma**. 400 (column 1), 300 (column 2), 200 (column 3) synaptic inputs asynchronously activated at μ_onset_ = 15*ms* with standard deviation σ_onset_ = 5*ms*.

In all setups, synchronous as well as asynchronous, calcium levels at the soma exclusively depend on whether or not an action potential is elicited. We see step increases in the calcium concentration with every action potential. Calcium diffusion, however, is only able to transport calcium within a very local vicinity of its original point of entry at active synapses. After termination of electrical signaling, calcium levels exponentially decay to equilibrium levels due to the activity of NCX and PMCA pumps. This shows a direct correspondence between synapse loss and somatic calcium levels through the number of action potentials elicited in a neuron. Sample evolution of membrane potential and calcium concentration at the soma (from the moderately asynchronous setting) for various levels of excitatory synapse loss are depicted in Figure 8.

### 3.2. Large-scale network simulations with detailed anatomy

#### 3.2.1. Parallel scaling

In order to test the parallel scaling properties of our network simulation implementation, we created six neocortical geometries containing 320, 640, 1280, 2560, 5120, and 10,240 neurons, respectively. The average number of compartments per neuron in the six geometries ranged from 574 to 586. We defined a random thalamic activation pattern, where synapse activation times and durations for the thalamic input synapses created by NeuGen were drawn from the same normal distribution for all geometries. We then performed one thousand time steps using 32, 64, 128, 256, 512, and 1024 processors of the Jülich supercomputer JUQUEEN on the geometries, respectively—thus in each simulation, a processor would be assigned approximately the same amount of work (“weak scaling”). We profiled the execution of the program to obtain the amount of time spent in the main components of the simulation. Table 5 shows the results. Leaving out the loading of the geometry into memory and its distribution to the involved processors (both are inherently serial), we achieve good scaling. The times spent for preparing the channel mechanisms and synapses before a time step, for assembling, for factorizing the matrix and applying the inverse remain approximately constant. As a typical network simulation will have more than 1000 time steps, the loading and distribution of the domain (which is only performed once, i.e., at the start of the parallel simulation) will have much less of an impact on scaling behavior than in this particular study. We thus demonstrated that our code is suitable to be used efficiently for simulations of large-scale networks of neurons.

**Table 5:** **Weak parallel scaling results obtained by code profiling**.

**Problem size (#neurons)**	**320**		**640**		**1280**		**2560**		**5120**		**10240**
**#Processors**	**32**	**×**	**64**	**×**	**128**	**×**	**256**	**×**	**512**	**×**	**1024**
Loading domain	12.8	2.08	26.6	2.03	54.1	2.09	113	2.17	246	2.29	562
Domain distribution	4.89	2.12	10.4	2.06	21.4	2.11	45.3	2.10	95.0	2.07	197
Determining step size	42.0	1.03	43.5	1.02	44.2	0.98	43.4	1.00	43.4	1.02	44.1
Preparing time step	52.9	1.01	53.5	0.99	53.2	1.02	54.2	1.02	55.5	1.13	62.5
Assembling system	325	1.02	330	1.03	338	0.97	329	0.99	327	1.02	333
Applying solver	24.0	1.03	24.8	1.01	25.0	0.94	23.4	1.04	24.3	0.92	22.3
Rest	6.41	1.90	12.2	0.33	4.00	2.18	8.7	2.05	17.8	1.13	20.1
Total time	468	1.07	501	1.08	540	1.14	617	1.31	809	1.54	1241
Total w/o load & distribute	450	1.03	464	1.00	465	0.99	458	1.02	468	1.03	483

*Timings show almost ideal scaling for setting up the system of equations as well as solving it. Loading and distributing the domain to the involved processors is inherently serial as it is done by a single processor in every simulation—however, the time percentage of the two tasks is much lower in typical simulations as they run much longer and loading and distributing is only performed once at the beginning. The remaining variance of the simulation runs is mainly due to differences in the quality of domain distribution. All time values in units of seconds*.

#### 3.2.2. Network connectivity affects network activity

When a neuronal network is created by NeuGen, synapses connect presynaptic axons to postsynaptic dendrites (if the involved neuron types allow this) where axon and dendrite are sufficiently close to each other (cf. Wolf et al., 2013). The maximal distance dist_synapse for which synapses are placed can be chosen by the user. This criterion, albeit not representative of an actual model of synaptogenesis (NeuGen does not reproduce neuronal growth, but only a fully grown state), might be considered as a parameterization of the agility of filopodia and growth cones during synaptogenesis (Munno and Syed, 2003). In any case, it has a direct effect on the connectivity properties of the network.

**Figure 9:**
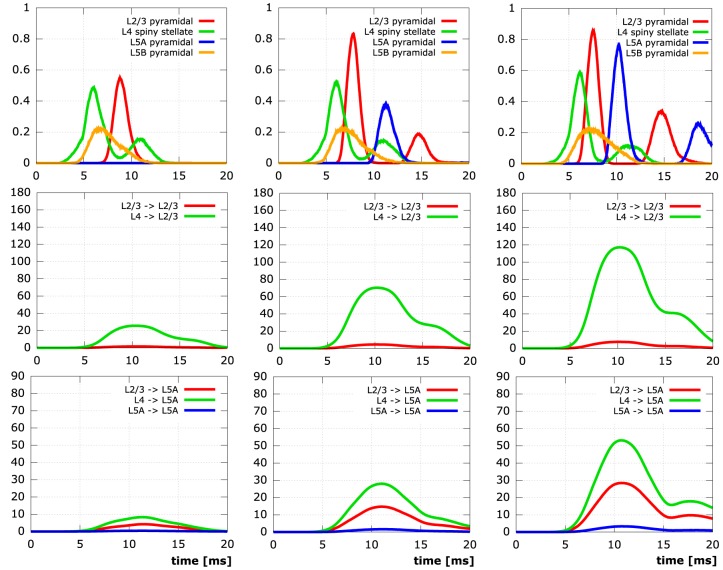
**Simulation results for networks of 10,000 neurons**. The columns contain time plots for networks created with a synapse creation distance of 2 µm, 3 µm, 4 µm (FLTR), respectively. Row 1: Relative number of active somata, i.e., *V* ≥ −45 mV, in different levels (L2/3 red, L4 green, L5A blue, L5B orange). Row 2: Number of active synapses at L2/3 pyramidal neurons originating from different levels (L2/3 red, L4 green). Row 3: Number of active synapses at L5A pyramidal neurons originating from different levels (L2/3 red, L4 green, L5A blue). Initial activation of L4 spiny stellate and L5B pyramidal cells by the same thalamic input pattern in all simulations.

We conducted simulations on five neocortical networks, each composed of the same 10,000 neurons, but with the connection distance ranging from 1 µm to 5 µm in steps of 1 µm. This resulted in networks with increasing numbers of synapses and connected neurons (Table 3). As previously described in Wanner (2007), we initialized network activity by depolarizing L4 spiny stellate cells via primary thalamic input synapses, activity then spread out through the cortical layers due to interconnecting synapses. Analysis of the time courses of the membrane potential at the somata in conjunction with activity data from the interconnecting synpases (Figure 9) reveals significant impact of the connectedness on the overall qualitative (and quantitative) behavior following the same thalamic input pattern in all five simulations.

In the least connected network, the number of synapses connecting thalamically activated L4 spiny stellate cells to L2/3 pyramidal cells (only 7.5 per L2/3 pyramidal cell on average) does not suffice to lead to the depolarization of a single L2/3 cell in the network. Obviously, this means there can be no active synapses connecting L2/3 to L5A and although there are also synapses connecting L4 to L5A directly, there is no activity in L5A, either. While in the network next in synapse number, considerable depolarization of layer 2/3 pyramidal neuron somata manifests itself due to 7.5-fold increase in average number of active synapses from L4 to L2/3, there is still practically no signal in L5A. Only in the networks created with synapse creation distance parameters ≥3 µm are action potentials elicited at the somata of L5A. The same networks exhibit the formation of a second action potential in some of the initially activated L2/3 somata, the two most connected networks also show the occurrence of a second action potential in some of the L5A cells. These second action potentials are the combined effect of (i) charge from previous synaptic inputs that has not yet been cleared and (ii) additional influx at the re-activated synapses. It is noteworthy that somatic activity in both L2/3 and L5A pyramidal cells peaks higher (L2/3: 0.0, 0.56, 0.84, 0.87, 0.87; L5A: 0.0, 0.0, 0.39, 0.78, 0.87) and earlier (L2/3: –, 8.8 ms, 7.9 ms, 7.6 ms, 7.6 ms; L5A: –, –, 11.4 ms, 10.2 ms, 9.7 ms) the more synaptic connections there are in the respective cortical layers.

**Figure 10:**
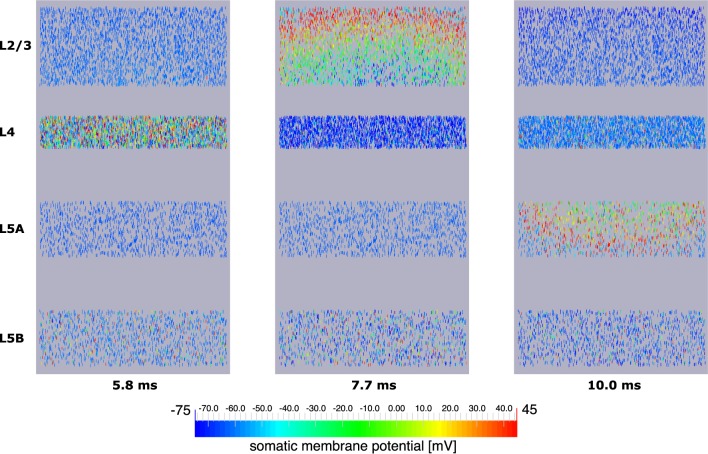
**Signal propagation through a neocortical slice of 10,000 neurons**. Only somata are visualized. Primary excitation of L4 spiny stellate cells (left) is followed by activation of somata of L2/3 pyramidal cells (middle), before the signal propagates to pyramidal neurons of layer 5A (right). Note how the depolarization of somata expands through layers 2/3 and 5A like a wavefront indicating increased signal run time to neurons more remote with respect to the signal origin.

Explicit influence of spatial extensions of the neural network can be identified in Figure 10: Somatic depolarization and hyperpolarization expands through the layers of L2/3 and L5A pyramidal cells like a wave, activating the neurons in the order imposed by the distance to the respective origin of that activation.

## 4. Discussion

In this paper we presented studies of electrical and biochemical signals in single cells and networks to investigate the interplay between synapse loss and signaling synchrony. A major focus was the anatomically realistic representation of cells and networks, for which a novel simulation toolbox NeuroBox was developed.

The synapse distribution studies on the layer 3 pyramidal cell from the rat neocortex show a significant impact of the activation pattern (in space and time) on the signal conductance capabilities of the cell. Two effects are apparent: (1) The more asynchronous the input signals are, the more spikes can be generated by this input—up to a point where the asynchrony begins to affect the likelihood of generating a single spike. (2) The more synchronous input signals are, the higher the cell's resilience is to synapse loss with regard to its capability of generating action potentials in response to synaptic input.

In the context of the study of synaptic input patterns, we also conducted simulations of calcium dynamics, including Ca^2+^ influx through synaptic AMPA-R channels as well as voltage-dependent calcium channels, NCX and PMCA pump mechanisms distributed throughout the membranes of dendrites and soma. Results showed that the somatic calcium concentration, key factor in the control of gene expression (Hardingham et al., 1997) and thus development and survival of cells, is directly coupled to the number of action potentials initiated in the cell, each action potential leading to a step increase in calcium levels. However, we neglected effects of internal calcium stores and also correct consideration of calcium buffers here. Especially the large amounts of calcium releasable through ryanodine and IP_3_ receptor channels in the membrane of the endoplasmic reticulum need to be taken into account in a detailed three-dimensional simulation in order to achieve a more accurate description of calcium signaling, possibly including calcium waves (cf. Berridge, 1998, among others). A method of coupling the one-dimensional simulation of the membrane potential to a detailed three-dimensional simulation of calcium signals has previously been developed by the authors (Grein et al., 2014) and may be applied here.

Using NeuGen for the generation of a neocortical column, we have shown that our implementation of a compartment model for the cable equation and trans-membrane current mechanisms in neural networks is adequate for large-scale applications and scales well with the number of neurons involved. It is reasonable to assume that simulations on even larger neural networks can successfully and efficiently be conducted on high-performance computers with the help of our implementation.

The neural network simulations we performed were very basic in nature. We only considered four of the diverse neuron types present in the neocortex. Unlike (e.g., Anderson et al., 2007; Vierling-Claassen et al., 2010; Neymotin et al., 2011), we did not take into account inhibitory synapses and their role in regulating cortical signal processing. Unlike the three aforementioned contributions, however, we created neural networks whose spatial resolution—about 500 compartments per neuron in our simulations as compared to 3, 16 and 1 in theirs—allowed for a realistic spatial positioning of synapses. We utilized the simple (yet not unreasonable) distance rule of NeuGen to create synapses instead of putting experimental projection data (as extensively reviewed for excitatory neurons by Feldmeyer, 2012) to good use. Incorporation of experimental findings into the existing framework, however, is not difficult. The addition of inhibitory synapses, for instance, is merely a question of re-parameterization in a preprocessing step. All that considered, our network simulations make it possible to examine the impact of intra- and trans-laminar synaptic connections on each level and can therefore serve as a valuable tool to decipher the functional role of detailed anatomy in cortical information processing.

With a focus on accessible workflow control that includes high-performance numerical methods, a modular neuroscientific repository and the option of including third-party tools, we developed the toolbox NeuroBox and used it to perform all simulations in this paper. NeuroBox is an open source project hosted on github with the intent to offer its full functional scope to a broad community. Visual workflow design and control through VRL-Studio makes NeuroBox projects easy to use and share with experts and non-experts alike. This feature is highly beneficial for rapid prototyping and offers an efficient pathway from *in silico* experiment design to full implementation thereof.

The possibility to integrate third-party tools, such as ImageJ, anatomical reconstructions (e.g., neuromorpho.org) and the automated import of NMODL models, integrates NeuroBox ideally into ongoing endeavors in the computational neuroscience field. Due to the modular design, this toolbox is easily extendible through various pathways discussed in Section 2 and thus can grow with continued research. As problem sizes typically increase alongside growing high-performance computing power, NeuroBox was built with links to UG 4, a general purpose package for solving partial differential equations. Advanced numerical methods with time and space adaptivity, error estimation and parallel communication layer advance the possibilities for solving anatomically realistic large-scale network problems.

## Author contributions

Cable equation modeled and implemented by MB and PG. Synapse handling designed and implemented by MB, SG, LR, MS. Neuron and network morphology preparation by SG. Network simulations carried out by MB and PG, synapse loss and calcium simulations by MB, PG, LR, and MS. GQ co-designed all methods and experiments and analyzed data. MB, SG, GQ, and MS wrote the manuscript.

## Funding

The work presented in this paper was funded by the BMBF (Bernstein Center for Computational Neuroscience Heidelberg/Mannheim) and the program for US-German collaborative research in Computational Neuroscience (01GQ1410B).

### Conflict of interest statement

The authors declare that the research was conducted in the absence of any commercial or financial relationships that could be construed as a potential conflict of interest.
