# Supplementary material for: Anatomically Detailed and Large-Scale Simulations Studying Synapse Loss and Synchrony Using NeuroBox
Source: Front Neuroanat. 2016 Feb 12;10:8. doi: 10.3389/fnana.2016.00008 (PMC4751272; doi:10.3389/fnana.2016.00008)
Supplement: Supplementary file 1 [file DataSheet1.pdf]

Listing 1. Example Lua script for a network simulation using UG4.

```lua
----------------------------------------------
-- This script sets up a network simulation. --
----------------------------------------------

-- load utility functions
ug_load_script("cable_util.lua")

-- choice of morphology
gridName = "testNetwork.ugx"

-- the following geometric subsets need to be defined in the network file
neededSubsets = {"Axon", "Dendrite", "Soma", "PreSynapseEdges", "PostSynapseEdges"}

-- init UG (for 3-dim coordinate space)
InitUG(3, AlgebraType("CPU", 1));

-----------------------------
-- biophysical model setup --
-----------------------------
-- equilibrium potential (in units of mV)
v_eq = -65.0

-- temperature in units of deg Celsius
temp = 37.0

-- cable equation (here: only potential, no ions)
CE = CableEquation("Axon, Dendrite, Soma, PreSynapseEdges, PostSynapseEdges", false)
CE:set_spec_cap(1e-5)       -- specific capacitance (units of 10^3 F/m^2)
CE:set_spec_res(1.5e6)      -- specific resistance (units of 10^-6 Ohm m)
CE:set_ek(-90.0)            -- reversal potential K (mV)
CE:set_ena(60.0)            -- reversal potential Na (mV)
CE:set_temperature_celsius(temp)

-- Hodgkin and Huxley channels --
-- specific membrane conductances (in units 10^6 S/m^2)
g_k_ax = 4.0e-4 -- axon
g_k_so = 2.0e-4 -- soma
g_k_de = 3.0e-5 -- dendrite

g_na_ax = 3.0e-2
g_na_so = 1.5e-3
g_na_de = 4.0e-5

HH = ChannelHH("", "Axon, Dendrite, Soma, PreSynapseEdges, PostSynapseEdges"
HH:set_conductances(g_k_ax, g_na_ax, "Axon, PreSynapseEdges")
HH:set_conductances(g_k_so, g_na_so, "Soma")
HH:set_conductances(g_k_de, g_na_de, "Dendrite, PostSynapseEdges")
CE:add_channel(HH)
```

```
-- leakage (needs to be precisely calibrated to attain the aspired
-- resting potential in each part of the network! -- in our case: -65mV everywhere)
tmp_fct = math.pow(2.3,(temp-23.0)/10.0)
leak = ChannelLeak("v", "Axon, Dendrite, Soma, PreSynapseEdges, PostSynapseEdges")
leak:set_cond(2.0e-4*tmp_fct, "Axon, PreSynapseEdges")
leak:set_rev_pot(-66.148458, "Axon, PreSynapseEdges")
leak:set_cond(1.0e-6*tmp_fct, "Soma")
leak:set_rev_pot(-30.654022, "Soma")
leak:set_cond(1.0e-6*tmp_fct, "Dendrite, PostSynapseEdges")
leak:set_rev_pot(-57.803624, "Dendrite, PostSynapseEdges")
CE:add_channel(leak)

-- synapses
syn_handler = NETISynapseHandler()
syn_handler:set_cable_equation(CE)

-- tell synapse handler where presynapses are located
syn_handler:set_presyn_subset("PreSynapse")

-- define an excitatory input pattern for primary synapses
-- activating the network
syn_handler:set_activation_timing(
    0.0,    -- average start time of synaptical activity in ms
    2.4,    -- average duration of activity in ms (10)
    0.0,    -- deviation of start time in ms
    0.0,    -- deviation of duration in ms
    1.2e-3) -- peak conductivity in units of uS
CE:set_synapse_handler(syn_handler)

----------------------------------------------------
-- handing the problem over to UG4 and running it --
----------------------------------------------------
-- reads the network file and distributes network to available processors
-- sets up approximation space for unknown membrane potential
-- sets up space and time discretizations for the given model
-- sets up a solver
ugEnv = setup_problem_ug4(CE, gridName, neededSubsets)

-- parameters steering the simulation
-- can be provided on the command line,
-- default value defined here is used otherwise
simParams = {}
simParams.v_init = v_eq
simParams.startTime = 0.0
simParams.dt = util.GetParamNumber("-dt", 0.02, "integration time step")
simParams.endTime = util.GetParamNumber("-endTime", 100.0,  "end time of simulation")
simParams.generateVTKOutput = util.HasParamOption("-vtk")
simParams.vtkFolder = util.GetParam("-outName", "simulation_output")
simParams.plotStep = util.GetParamNumber("-plotStep",   dt,     "plotting interval")

-- runs the specified simulation
run_simulation(ugEnv, simParams)
```
